# Rotavirus Antagonism of the Innate Immune Response

**DOI:** 10.3390/v1031035

**Published:** 2009-11-24

**Authors:** Michelle M. Arnold, John T. Patton

**Affiliations:** Laboratory of Infectious Diseases, National Institute of Allergy and Infectious Diseases, National Institutes of Health, 50 South Drive MSC 8026, Room 6314, Bethesda, MD 20892-8026, USA; E-Mail: arnoldm@niaid.nih.gov

**Keywords:** rotavirus, NSP1, interferon regulatory factor, IRF

## Abstract

Rotavirus is a primary cause of severe dehydrating gastroenteritis in infants and young children. The virus is sensitive to the antiviral effects triggered by the interferon (IFN)-signaling pathway, an important component of the host cell innate immune response. To counteract these effects, rotavirus encodes a nonstructural protein (NSP1) that induces the degradation of proteins involved in regulating IFN expression, such as members of the IFN regulatory factor (IRF) family. In some instances, NSP1 also subverts IFN expression by causing the degradation of a component of the E3 ubiquitin ligase complex responsible for activating NF-κB. By antagonizing multiple components of the IFN-induction pathway, NSP1 aids viral spread and contributes to rotavirus pathogenesis.

## 1. Introduction

**Figure 1.**
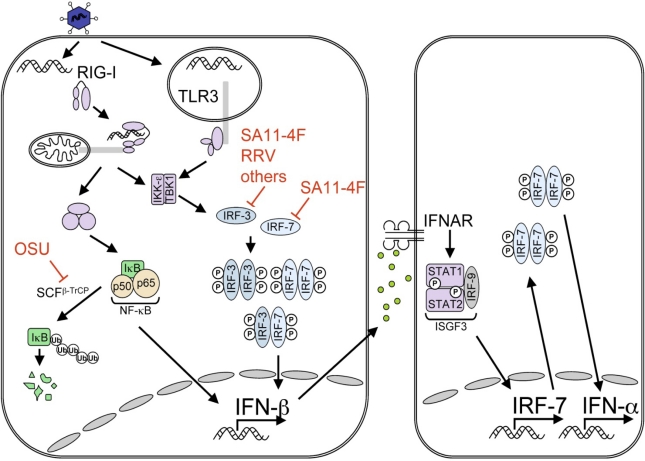
Summary of NSP1 inhibition of the innate immune response pathway. Activation of dsRNA sensors in the cytoplasm (such as RIG-I) or bound to membranes (such as TLR3) stimulates pathways that result in the phosphorylation of IRF3 and IRF7 by IKK-ɛ and TBK1. Phosphorylation allows for the formation of IRF3 homodimers, IRF7 homodimers, or IRF3/IRF7 heterodimers. RIG-I activation also results in the ubiquitination (Ub) of IκB by the E3 ligase Skp1/Cul1/F-box complex SCF^β-TrCP^ and subsequent degradation by the proteasome. Degradation of IκB frees the NF-κB complex (p50 and p65), which moves to the nucleus. The binding of NF-κB and IRF dimers to the IFN-β promoter results in transcription and secretion of IFN-β, which binds to type I IFN receptors (IFNAR). IFNAR signals the ISGF3 complex, consisting of STAT1, STAT2, and IRF9, to produce additional IRF7 and type I IFNs, thus amplifying the IFN response. The NSP1 protein from different RVs has a range of activities. Some RV NSP1 proteins inhibit the type I IFN response by degrading IRF3 and IRF7, while at least one NSP1 prevents type I IFN induction by degrading β-TrCP and preventing the nuclear translocation of NF-κB.

The innate immune system provides an immediate mechanism of suppressing the replication and spread of viruses. The production of type I interferon (IFN), including IFN-β and IFN-α subtypes, is critical to an effective innate immune response. Type I IFNs activate the expression of hundreds of IFN-stimulated genes (ISGs), which play critical roles not only in the innate immune response, but also in B-cell and T-cell activation, cell cycle regulation, and apoptosis [1,2]. ISGs can interfere with viruses at various stages of their replicative cycle. To counteract the innate immune response, most viruses have evolved mechanisms to subvert IFN signaling, including rotaviruses (RVs), members of the *Reoviridae* family. RV antagonizes the IFN induction pathway through the action of the nonstructural protein NSP1, which induces the proteasomal degradation of one or more transcription factors necessary for the efficient expression of type I IFN (Figure 1).

### IFN Response to Viral Infection

The IFN response can be triggered in virus-infected cells by the interaction of viral nucleic acids or proteins with pattern-recognition receptors (PRRs). Two families of PRRs have been identified as primary sensors of viral infection: the transmembrane TLRs (Toll-like receptors) and the cytoplasmic pathogen detectors, including the RNA sensors RIG-I (retinoic acid-inducible gene I) and MDA5 (melanoma differentiation-associated gene 5), and the DNA sensor DAI (DNA-dependent activator of IFN-regulatory factors) [3–5]. The interaction of viral nucleic acids with PRRs leads to the activation of IFN regulatory factors (IRFs) and nuclear factor (NF)-κB, which translocate from the cytoplasm to the nucleus where they stimulate transcription of type I IFN genes. Collectively, IFN expression requires the participation of several transcription factors, providing viruses many potential targets for antagonizing the IFN signaling pathway [6].

Members of the IRF family of proteins include IRF3 and IRF7, key regulators of type I IFN expression [7]. IRF3 is constitutively expressed in cells where it accumulates at elevated levels in the cytoplasm. In contrast, IRF7 is present in most cells at very low levels, and its expression is amplified by type I IFN [7,8]. Both IRF3 and IRF7 reside in an inactive state in the cytoplasm and are activated by phosphorylation of residues in the C-terminal regulatory region by the kinases TBK1 or IKK-ɛ [9,10]. Phosphorylated IRF3 and IRF7 form homodimers or heterodimers, which then translocate to the nucleus. IRF3 homodimers, IRF7 homodimers, and IRF3/IRF7 heterodimers have differential effects on the expression of type I IFN gene-family members [11–13]. IRF3, in cooperation with other transcription factors, is an activator of IFN-β and a few IFN-α subtypes. IRF7 has broader effects than IRF3, promoting the expression of even higher levels of IFN-β and a greater number of IFN-α subtypes. Maximal expression of type I IFN is achieved through the combined actions of IRF3 and IRF7 [9,12,14,15]. Other members of the IRF family, such as IRF5, are known to play important roles in the development of antiviral responses. However, their contribution to effective IFN expression is not well understood and may vary depending on host species [4].

Secreted type I IFNs bind to type I IFN receptors (IFNAR) and induce the formation of IFN-stimulated gene factor 3 (ISGF3), a heterotrimeric complex consisting of signal transducer and activator of transcription (STAT) 1, STAT2, and IRF9. ISGF3 translocates to the nucleus and stimulates transcription of ISGs, including IRF7. Thus, in most cell types, IRF7 levels are increased through positive feedback by type I IFNs [13,14]. However, IRF7 is constitutively expressed at high levels in plasmacytoid dendritic cells (pDC), the primary source of type I IFN in response to infection [16]. IRF7 has been shown to be crucial for virus-induced IFN-β and IFN-α expression in both pDC and non-pDC cell lines [17].

NF-κB is also required for inducing transcription of the IFN-β gene, but not for IFN-α genes [11,18]. NF-κB subunits are held inactive in the cytoplasm through an interaction with inhibitors of κB (IκB). Phosporylation of IκB by IκB kinases (IKKα/β) results in its ubiquitination by the E3 ligase Skp1/Cul1/F-box complex SCF^β-TrCP^, and subsequent degradation by the proteasome [19]. IκB degradation releases the NF-κB heterodimer p50/p65, which allows it to translocate to the nucleus for promoter binding and transcription of genes containing NF-κB response elements.

## 2. Rotavirus Biology

RV is one of several genera of segmented double-stranded (ds) RNA viruses within the family *Reoviridae*. RV is a primary cause of severe dehydrating gastroenteritis in children under 5 years of age, causing approximately 500,000 deaths worldwide each year [20]. Though the global burden of diarrheal disease has been decreasing, children living in developing countries are more likely to die from RV infection [21]. The introduction of vaccines in some countries in North and Latin America, Europe, and Australia has reduced the number of cases of severe diarrhea and deaths. These vaccines are currently not available or not affordable in the poorest countries, and their efficacy may be suboptimal in impoverished settings. This latter issue may stimulate renewed efforts to modify existing or to develop a new generation of more efficacious vaccines. One potential strategy for developing new vaccine candidates is to engineer human RVs with a weakened ability to antagonize host IFN induction pathways.

### 2.1. Replication

**Figure 2.**
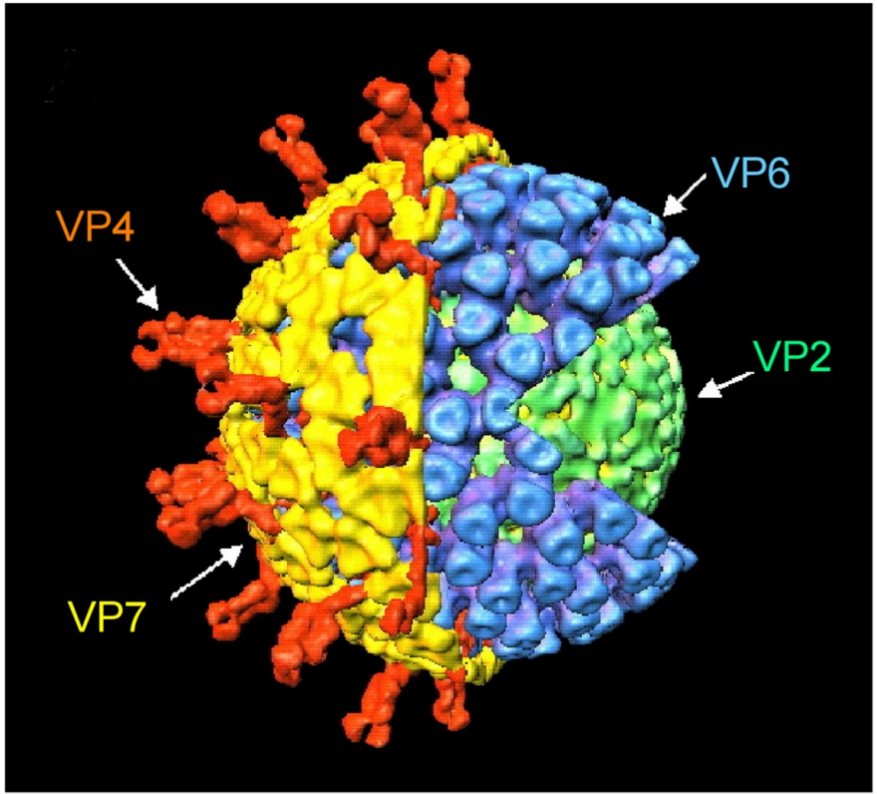
Three-dimensional structure of the RV virion. Genomic dsRNAs are associated with the RdRP VP1 and the capping enzyme VP3 on the interior side of the VP2 (green) core shell. An intermediate layer of VP6 (blue) surrounds the core. The outer layer of the virion is comprised of a VP7 capsid (yellow) with VP4 spikes (orange) protruding through this shell, completing the infectious virion. Figure was kindly provided by B.V.V. Prasad (Baylor College of Medicine) and details are given in [22,25,30].

The RV virion is an icosahedral particle composed of three concentric layers of protein that encapsidate 11 segments of dsRNA (Figure 2) [22,23]. The virion core includes the inner shell protein VP2, small amounts of the viral RNA-dependent RNA polymerase (RdRP) VP1 and the mRNA-capping enzyme VP3, and the segmented dsRNA genome [24–26]. Cores surrounded by a protein layer formed by VP6 represent double-layered particles (DLPs). In the infected cell, DLPs are transcriptionally active and are responsible for the synthesis of the 11 viral mRNAs [27,28]. The outer protein layer of the capsid is comprised predominantly of the glycoprotein VP7. Protruding from the VP7 layer are spikes formed by the protease-sensitive attachment protein VP4. VP4 and VP7 induce neutralizing antibody responses in the infected host, and are the basis for immunological protection against subsequent disease [29].

RV mRNAs contain methylated 5′ caps but lack 3′ poly(A) tails [31,32]. Translation of viral mRNAs yields six structural proteins (VP1-VP4, VP6-VP7) and six nonstructural proteins (NSP1-NSP6). In addition to serving as templates for protein synthesis, RV mRNAs are also used by the viral RdRP as templates for the synthesis of genomic dsRNAs [33,34]. Genome replication and packaging occur in coordination with the assembly of cores, which takes place in cytoplasmic inclusion bodies known as viroplasms [26,35]. DLPs are also assembled in association with viroplasms, and then migrate to the endoplasmic reticulum (ER), where via budding the particles acquire the outer capsid VP7-VP4 protein shell [36]. The predominant mechanism of virus release from RV-infected cells is by lysis, though there is evidence that a subpopulation of virions may be released from the apical membrane of polarized cells by a non-lytic mechanism [37,38].

The segmented character of the RV dsRNA genome allows distinct strains of the virus to exchange (reassort) genetic material during coinfection [39]. Such reassortment provides a mechanism for the rapid evolution of the virus and possibly the emergence of new antigenically-distinct virus strains. Selective pressures placed on individual viruses in nature may result in the maintenance of certain genome constellations encoding viral proteins that function optimally as a set, limiting the extent of reassortment.

### 2.2. Classification

RVs are classified into seven distinct groups, A to G, based on the antigenicity of the VP6 protein. Those in group A represent the major causative agents of acute gastroenteritis in humans and animals. Human group A RVs are divided into three distinct genogroups based on overall similarity to the three prototype strains: Wa, DS-1, and AU-1 [40]. Animal group A RVs are similarly divided into genogroups, though the divisions are more complex, with some genogroups including strains of RVs isolated from different animal species [41,42]. The antigenic and sequence characteristics of the outer capsid proteins VP7 (glycoprotein) and VP4 (protease-sensitive) have been used to classify group A RVs into G and P serotypes and genotypes [43]. Thus far, 22 G genotypes and 31 P genotypes have been defined [41,44–47]. Recently, a comprehensive sequence-based classification and nomenclature system was defined for RV, allowing each genome segment to be assigned a particular genotype [41].

### 2.3. Pathogenesis

RV infects mature differentiated enterocytes near the tips of the villi of the small intestine, causing mild inflammation, severe villus blunting, and malabsorption in the small intestine [48,49]. These effects contribute to the severe watery diarrhea and vomiting that are characteristic of RV disease, which in their most extreme manifestations can lead to death due to dehydration. Recent studies indicate that RV infections in children and animal model systems are not always limited to the intestine, but in some cases can spread beyond the gut, causing a transient viremia and a more systemic infection that involves the liver, lung, kidney, spleen, and mesenteric lymph nodes [49–54].

### 2.4. RV Host Range Restriction

RVs are highly infectious in their natural (homologous) hosts, with even small amounts of virus (≤1 plaque-forming units) capable of causing productive infection and severe disease. In contrast, successful RV infection of a heterologous host typically requires a concentrated-virus inoculum, yields little progeny virus, and is asymptomatic. The characteristic attenuated phenotype of RVs in their heterologous hosts has provided the foundation for the creation of human vaccines from bovine (RotaTeq, Merck) and rhesus (RotaShield, Wyeth) strains of RVs [21].

The basis for RV host range restriction is not fully understood, but analysis of the growth and virulence characteristics of RV reassortants has identified several gene products that may be involved. The outer capsid proteins VP4 and VP7 have been implicated in species-specific RV growth due to their roles in virus attachment and entry [55–57]. Moreover, studies of reassortants indicate that the RV enterotoxin protein NSP4, host translation inhibitor protein NSP3, and RNA capping enzyme VP3 influence the growth, spread, and virulence of viruses in different animal species [36,53,54,57]. A study investigating viral genes of murine RVs responsible for virulence in a mouse model obtained strong evidence that NSP1 has a role in viral pathogenesis [55]. Notably, the VP3, VP4, VP7, NSP1 and NSP4 proteins each show a degree of species-specific sequence variation, consistent with the idea that the activities of these proteins may vary depending on host cell type and thus provide a partial basis for host cell restriction.

### 2.5. Role of IFN in RV infection

Rotavirus infection stimulates the production of type I and II IFN in children and animals [58–60]. Elevated IFN-α is present both in the serum and stool of the infected host [59,61,62]. IFN-α administered prior to infection reduces RV-associated diarrhea in bovine and porcine models of infection, indicating that IFN restricts RV growth in some animals [63,64]. In contrast, IFN pretreatment of neonatal mice has little or no effect on the course of diarrhea or virus shedding caused by infection with some strains of RV [65]. More recent studies have shown that homologous and heterologous strains of RV differ in the ability to spread in mice deficient in IFN signaling. These results raise the possibility that the host IFN response, and the capacity of the virus to overcome it, may play a role in limiting intestinal replication and extraintestinal spread [66].

Type I IFNs also inhibit the growth of RVs in cell culture, although RV is not as sensitive to IFN as vesicular stomatitis virus (VSV) [67,68]. The human intestinal epithelial cell lines HT29 and Caco-2 cells are resistant to RV infection when pretreated with IFN-α [69]. HT29 cells do not express IFN-β mRNA following RV infection with the simian strain RRV, though evidence of NF-κB and NF-κB-dependent chemokine expression is apparent [70–72].

### 2.6. Properties of NSP1

**Figure 3.**
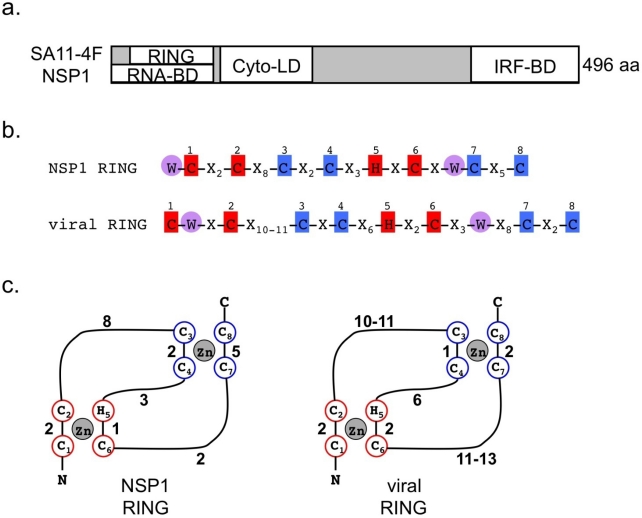
**(a)** Schematic of general location of structural and functional domains in SA11-4F NSP1; RING finger domain (RING); RNA-binding domain (RNA-BD); cytoplasmic localization domain (Cyto-LD); IRF-binding domain (IRF-BD). **(b)** Comparison of the consensus sequence of the RING finger domain of group A and C RV NSP1 and the viral RING domain. Conserved residues proposed to bind to first zinc ion highlighted in red boxes and conserved residues proposed to bind to second zinc ion highlighted in blue boxes. Numbers above boxes correspond to sequential numbering of metal ligands. Conserved tryptophan residues highlighted in violet circles. **(c)** Predicted cross-brace organization of the RING finger domain of NSP1 and comparison to the known viral RING domain organization. Subscript numbers correspond to sequential numbering of metal ligands, and interspersed numbers correspond to the residues between metal ligands.

NSP1 (referred to as NS53 or VP5 in early literature) is the ∼58-kDa protein product of RV genome segment 5 (Figure 3A). The protein accumulates at low levels in the cytoplasm of infected cells, possibly in association with the cytoskeleton [73–75]. The gene 5 RNA of group A RVs is unique in that its total length, as well as that of its open reading frame (ORF), can vary considerably among different virus strains. As a result, the NSP1 protein ranges in size from 486 to 496 amino acids for mammalian RV isolates, to as much as 577 amino acids for avian isolates. The amino acid sequence of NSP1 also differs significantly from strain to strain, more so than for any other RV protein. Notably, NSP1 sequence identities for mammalian RVs fall below 40%, and below 10% when the NSP1 sequences of mammalian and avian strains are compared.

Although divergent in primary sequence, the conservation of proline residues among NSP1 proteins suggests that they share similar higher order structures [76–78]. The N-terminal sequence of NSP1 is more conserved than that of the C terminus, and the N terminus includes the cysteine-rich motif, C-X_2_-C-X_8_-C-X_2_-C-X_3_-H-X-C-X_2_-C-X_5_-C (where X is any amino acid), which is common to all RV group A and C NSP1 proteins [75]. Early studies described the cysteine-histidine pattern as two zinc fingers, although recent studies have suggested this region is organized in a manner characteristic of those found in RING finger proteins (Figure 3B) [79,80]. NSP1 binds to zinc, but it has not been definitively demonstrated that the putative RING domain mediates this activity [81]. NSP1 also binds viral single-stranded (ss) RNA, and the RING domain is essential for this activity [74,81,82]. Mapping studies suggest that NSP1 recognizes the 5′-end of viral mRNAs, although the specific target of the binding activity has not been identified [74]. The role of the RNA-binding activity of NSP1 remains undefined.

Two lines of evidence indicate that NSP1 is not essential for RV replication. First, reducing NSP1 expression with gene-specific small-interfering RNAs during infection does not inhibit viral protein expression, gene synthesis, or virion assembly [35]. Secondly, RV variants have been isolated that encode C-truncated forms of NSP1, yet these viruses continue to replicate efficiently [82–85]. The most extreme example of an NSP1-defective variant is the bovine isolate A5-16, which encodes a truncated NSP1 that contains only the first 40 amino acids of the wild-type protein and therefore lacks the conserved zinc finger region [83,85]. This and other RV variants with a C-truncated NSP1 grow to titers similar to their wild-type parents in highly permissive MA104 cells [82,84]. However, in cells that are generally less permissive for RV replication (such as Caco-2 and FRhL_2_), wild-type RVs grow to titers that are at least one-log greater than NSP1-defective RVs, suggesting a role for NSP1 in enhancing cell-type specific viral replication [86].

Rotaviruses encoding wild-type NSP1 form plaques that are considerably larger than those of RVs encoding C-truncated NSP1, suggesting that NSP1 is linked to the efficient cell-to-cell spread of the virus [83–85,87]. For example, SA11-5S, a simian RV variant that expresses a C-truncated NSP1 that lacks the last 17 amino acids of the full-length protein, produces plaques that are roughly half the size of those produced by its parental virus SA11-4F [84]. The small plaque size of NSP1-defective RVs is consistent with a role for NSP1 in subverting IFN-dependent pathways that would otherwise impede the successful movement of RV from infected to uninfected cells.

## 3. Interaction of NSP1 with Transcription Factors of the IFN Induction Pathway

### 3.1. NSP1-Induced Degradation of IRF Proteins

The first evidence of an interplay between NSP1 and the innate immune system came from a yeast two-hybrid assay designed to identify proteins in a cDNA expression library of simian MA104 cells that interacted with NSP1 [88]. The analysis revealed that NSP1 of diverse origins (bovine B641 and murine EW RVs) bound to IRF3. The association of NSP1 and IRF3 during RV infection was confirmed by co-immunoprecipitation assays performed with human Caco-2 cells infected with a simian RV (SA11-4F) [89]. Derivative experiments revealed that the C-terminal one-third of NSP1 was sufficient for IRF3 interaction and that the deletion of a small C-terminal portion from NSP1 prevented IRF3 binding [88,89].

Infection of Caco-2 cells with SA11-4F reduced IRF3 to levels that were nearly undetectable. In contrast, infection with SA11-5S, which expresses a C-truncated NSP1, led to little decrease in IRF3 levels [89]. This result correlated expression of intact NSP1 with the capacity of RV to induce the degradation of IRF3. Direct evidence for NSP1-mediated degradation of IRF3 was provided by transient expression assays in which wild-type NSP1 or C-truncated NSP1 was co-expressed along with IRF3. Wild-type NSP1, but not the C-truncated form, induced IRF3 degradation [86]. Treatment with the proteasome inhibitor MG132 prevented NSP1-mediated degradation of IRF3, suggesting that IRF3 turnover is dependent on proteasome activity [89,90]. The ability of NSP1 to induce IRF3 degradation has been confirmed with multiple strains of RV in a variety of cell types (Table 1) [86,89–91].

Ultimately, NSP1-induced degradation of IRF3 precludes activation and nuclear accumulation of this transcription factor, which has a primary role in IFN-β expression. The importance that NSP1 has on IFN-β expression has been assessed using a bioassay with a green fluorescent protein (GFP)- expressing recombinant VSV (VSV-GFP). VSV growth, as measured by GFP fluorescence, is strongly inhibited by the presence of IFN in cell culture media. Studies using the bioassay system showed that VSV-GFP grows well in media taken from cells infected with wild-type RVs (SA11-4F, 30-19, and UK strains), demonstrating that RVs can suppress IFN expression [86]. In contrast, media recovered from cells infected with RV encoding a C-truncated NSP1 inhibited VSV-GFP growth, consistent with failure of its NSP1 to degrade IRF3. Importantly, the addition of a neutralizing anti-IFN-β antibody to the media recovered from cells infected with the NSP1-defective RV restored VSV-GFP growth. These studies are supported by experiments measuring levels of secreted IFN-β in RV-infected cells, and provide evidence that intact NSP1 is a significant antagonist of IFN-β expression [86,91].

The importance of the putative RING domain of NSP1 on IRF3 degradation has been addressed by mutagenesis. Specifically, the mutation of conserved cysteine and histidine residues in the RING weakened interactions between NSP1 and IRF3 in yeast two hybrid assays and prevented NSP1 from inducing IRF3 degradation in co-expression assays [90]. These findings raise the possibility that the RING domain is essential for the activity of NSP1, much like the RING domains of some classes of E3 ubiquitin ligases. However, it is possible that mutation of the RING domain caused such severe misfolding of NSP1 that a functionally-independent distant region of the protein involved in IRF3 degradation was rendered inactive.

Members of the IRF family share multiple structural features: all have an N-terminal DNA-binding domain that recognizes a consensus promoter sequence, several have a C-terminal serine-rich activation domain that is subject to phosphorylation, and others have a protein interaction domain that directs IRF dimerization [11]. These shared features raise the possibility that by interacting with one of the common domains, NSP1 can degrade members of the IRF family in addition to IRF3.

Indeed, transient expression assays have confirmed that the NSP1 product of the SA11-4F strain not only degrades IRF3, but also IRF5 and IRF7 through a proteasome-dependent process [86]. Thus, NSP1 represents a broad-spectrum antagonist of the IFN induction pathway. The biological significance of such broad activity is not clear, but the ability of NSP1 to induce the degradation of IRF7 suggests that some RVs may be particularly well suited to infect specialized cells, such as pDCs, that constitutively express IRF7. RVs can replicate in a minor proportion of pDCs, providing a means by which RVs can possibly escape the gut and give rise to a systemic infection [93]. Importantly, since pDCs are a primary source of IFN-α in the infected host, the capacity of RVs to infect these cells and degrade their IRF7 component may represent a particularly effective mechanism of down-regulating IFN-α expression in and around the gut. IRF5 is primarily detected in certain specialized cell types (e.g., B cells and DCs) [94]. The ability of NSP1 to degrade this transcription factor provides additional evidence that NSP1 assists the virus in infecting specialized cells that allow trafficking beyond the gut.

### 3.2. NSP1 Inhibition of the NF-κB Pathway

Numerous RV strains encoding full-length NSP1 have been shown to degrade IRF3 (Table 1) [86,89–92,95]. Surprisingly, the porcine RV strain OSU was shown to inhibit the expression of IFN-β, but failed to cause IRF3 degradation. In fact, IRF3 was activated and accumulated in the nucleus of OSU-infected cells. Further analysis has shown that OSU NSP1 can bind to IRF3, but less efficiently than B641 NSP1, suggesting a weak or unstable interaction incapable of triggering IRF3 degradation [90,96]. Because IFN-β expression requires not only activated IRF3, but also activated NF-κB, Graff *et al.* [96] examined the fate of NF-κB in OSU-infected cells. In uninfected cells, NF-κB is retained in the cytoplasm due to its interaction with IκB-α. Activation of NF-κB requires the interaction of phosphorylated IκB-α with the substrate recognition protein β-transducin repeat containing protein (β-TrCP) of the cellular E3 ubiquitin ligase complex SCF^β-TrCP^. This interaction results in the degradation of IκB-α, which then allows nuclear translocation of NF-κB [19]. OSU NSP1 was shown to induce the degradation of β-TrCP, which in turn prevents the degradation of IκB-α. As a result, IFN-β gene expression is not upregulated in OSU-infected cells due to the absence of activated NF-κB in the nucleus. The study by Graff *et al.* [96] also suggests that some NSP1 proteins may have multiple specificities, resulting in the degradation of both IRF3 and β-TrCP. The targeted degradation of a cellular F-box protein required for E3 ligase substrate recognition is a novel IFN evasion strategy not described previously for any other virus [96].

Additional studies have indicated that some strains of RV (human Wa) stimulate the phosphorylation of NF-κB. However, TNF-α-induced nuclear translocation and NF-κB-driven gene expression are inhibited during infection. Similarly, some strains (Wa) activate STAT1 by inducing phosphorylation, but IFN-α-induced STAT1 and STAT2 nuclear translocation are inhibited by RV infection. The mechanism by which RV brings about these phenomena remains unresolved, but this activity is not due to the expression of NSP1 alone [97].

### 3.3. Host Cell Dependence of NSP1 Activity

The neonatal mouse has been used extensively as an animal model system for identifying viral and host factors that influence RV growth, spread, and pathogenesis. Neonatal mice infected with many homologous (murine EC, EW, EDIM, ETD) and some heterologous (simian RRV) RVs develop diarrheal disease, but only during the first two weeks of life. Afterwards, these viruses cause asymptomatic infections in the mouse [21]. Early studies showed that treatment of neonatal mice with murine type I IFN prior to or after infection with EC RV had no effect on viral replication or diarrhea. This established that RVs have mechanisms for overcoming IFN-dependent antiviral responses. Further support for this idea came from the observation that diarrhea and virus shedding were not significantly reduced in IFN type I or type II receptor knockout (KO) mice [65].

Infection of the sucking mouse with some homologous RV strains (e.g., EC) leads not only to an enteric infection, but also to a systemic infection with the virus replicating in several internal organs. In addressing factors affecting virus spread, Feng *et al*. [66] found that enteric and systemic replication of the EC virus did not differ between wild-type and IFN signaling-deficient mice (IFN-α/β/γ receptor KO or STAT1 KO). However, enteric and systemic replication of a heterologous simian strain (RRV) was significantly increased in mice deficient in type I and type II IFN signaling (IFN-α/β/γ receptor KO or STAT1 KO) [66]. In adult mice, STAT1 deficiency was associated with increased viral shedding of the heterologous simian strain RRV [98]. These results suggest that IFN signaling has a role in modulating RV infection, but that some RV strains can antagonize this effect.

Additional studies using primary mouse embryonic fibroblasts (MEFs) found that the replication of some heterologous RV strains (UK, NCDV, and OSU) was restricted in wild-type MEFs, but not in IFN signaling-deficient (IFN-α/β/γ receptor KO or STAT1 KO) MEFs. However, homologous murine strains and the heterologous simian strain RRV replicated to similar titers in wild-type and IFN signaling-deficient MEFs. By analyzing a panel of reassortant viruses, the ability of RRV to replicate efficiently in wild-type MEFs was mapped to the NSP1 gene. Strains that replicated well were able to degrade endogenous IRF3 in wild-type MEFs, thereby inhibiting IFN-β secretion [91]. In contrast, heterologous strains that replicated poorly in wild-type MEFs failed to degrade IRF3 and stimulated the secretion of IFN-β. The production of IFN-β was dependent on viral replication, which triggered phosphorylation of IRF3. Though replication was restricted in wild-type MEFs, these strains grew well in IFN signaling-deficient MEFs. Only NSP1 was associated with a differential growth advantage in the presence of an intact IFN signaling system [91]. These results suggest strain-specific differences exist in NSP1-mediated degradation of IRF3 and suppression of IFN-β secretion. Bovine UK RV, one of the RV strains with restricted replication in MEFs that was unable to degrade endogenous murine IRF3, was also unable to degrade ectopically expressed murine IRF3 in simian Cos7 cells. This was not due to an inherent inability to degrade IRF proteins in general, as this strain was able to degrade endogenous simian IRF3 and ectopically expressed human IRF3 in MEFs [92]. Studies on the abilities of various heterologous and homologous strains of RV to replicate in MEFs continue to provide greater understanding into the potential role of NSP1 in RV host range restriction and cell tropism.

### 3.4. NSP1 as an E3 Ubiquitin Ligase

NSP1 has several characteristics that suggest it may function as a viral E3-ubiquitin (Ub) ligase. Perhaps central among these is that NSP1 contains a RING domain similar to that of some E3 ligases and that the protein induces the proteasomal degradation of target proteins. In addition, NSP1 is itself susceptible to proteasomal degradation, and treatment with proteasome inhibitors leads to an increased accumulation of NSP1 [80,90,92]. The degradation of NSP1 is likely mediated by polyubiquitination, as inhibition of the E1 Ub-activating enzyme activity enhances NSP1 accumulation [92].

Ubiquitination places signals on proteins that cause their redistribution in the cell or degradation by the proteasome. Polyubiquitination of a protein occurs through a cascade of events, whereby an E1 Ub-activating enzyme transfers the Ub moiety to an E2 Ub-conjugating enzyme. The E2 then interacts with one of two classes of E3 Ub ligases, which differ markedly in the mechanism by which they direct the ubiquitination of target proteins. The HECT domain-containing E3 Ub ligases undergo transient ubiquitination by E2 ligases, and then transfer the Ub moiety onto the target protein. In contrast, the RING domain-containing E3 ligases act as an adaptor between the target protein and the E2 ligase, which directly transfers the Ub moiety onto the target.

NSP1 contains a conserved cysteine-histidine sequence in its N-terminal region proposed to form zinc fingers [75,79]. Subfamilies of zinc finger proteins are generally classified by the order of the zinc-coordinating cysteine (C) and histidine (H) residues that serve as metal ligands. The classic RING finger domain consists of a signature C3-H-C4 motif, while the classic PHD motif is C4-H-C3 [99]. Some viral immunomodulatory proteins, such as Kaposi’s sarcoma herpesvirus K3 and K5, which were initially classified as PHD proteins according to their C4-H-C3 motif, have more recently been described as variants of the RING domain family, termed viral RING (vRING) [100]. These vRING proteins possess ubiquitin ligase activity, whereas classically defined PHD proteins do not. Additional criteria used to classify PHD domains include the presence of a tryptophan residue, which is positioned two amino acids upstream of metal ligand 7. The vRING domain, however, possesses two invariant tryptophan residues, which are located one amino acid downstream of metal ligand 1 and three amino acids upstream of metal ligand 6. Group A RV NSP1 proteins possess a C4-H-C3 core motif similar to that of PHD and vRING domains (Figure 3B, C). This region of NSP1 includes two highly conserved tryptophan residues; yet neither matches the positioning of the conserved PHD-specific tryptophan residue. Instead, the NSP1 tryptophan residues are positioned similar to those of vRING proteins. The first tryptophan of the NSP1 RING domain is one residue upstream of metal ligand 1 while the second is two residues upstream of metal ligand 6. An additional feature of vRING proteins is the predicted presence of transmembrane helices that direct these ubiquitin ligases to their targets, membrane-bound MCH class I proteins; however, this feature is not shared by NSP1. Based on sequence analysis, NSP1 shares homology with vRING proteins. Based on functional analysis, NSP1 induces the proteasomal-dependent degradation of IRF family members. Together, the sequence and function of NSP1 support the hypothesis that the protein functions as an E3 ubiquitin ligase. However, direct evidence in support of this has yet to be obtained.

### 3.5. NSP1 Similarities to Other Viral Proteins

Viruses use a diverse array of approaches to counteract the host IFN-signaling pathway (see [101] and [102] for review). Viruses can disrupt nearly every stage of the IFN response including early dsRNA signaling events (e.g., RIG-I, MDA5), the IFN receptor-signaling pathway (e.g., JAK, STAT), or specific ISG products (e.g., PKR or MxA). Like RVs, several other viruses suppress type I IFN expression by interfering with the IRF3 pathway, though the mechanisms of IRF3 antagonism vary. The human papillomavirus 16 protein E6 binds to IRF3 preventing transcription of IFN-β. However, the binding of E6 to IRF3 does not induce its ubiquitination or proteasomal degradation [103]. Classical swine fever virus (CSFV) downregulates the expression of IRF3, and the viral protein N^pro^ mediates this effect [104]. N^pro^ also associates with IRF3 and induces its degradation in a proteasome-dependent manner [105]. This is the same mechanism used by the related virus bovine viral diarrhea virus (BVDV) [106,107]. However, IRF7 expression does not appear to be affected by N^pro^ [105]. Characterization of N^pro^ shows that it is a zinc-binding protein, and that the zinc-binding site is required for IRF3 degradation and inhibition of IFN-β production. N^pro^ contains a metal binding TRASH (trafficking, resistance and sensing of heavy metals) motif at its C terminus, rather than the RING-finger motif typical of E3 ubiquitin ligases [108]. This mechanism of IRF3 inhibition is similar to the activity of RV NSP1, though it does not appear to be as broadly active as NSP1.

## 4. Conclusions and Perspectives

Although the NSP1 proteins of different RV strains can vary in their activities, it is clear that NSP1 is an antagonist of the type I IFN induction pathway. Of the types of NSP1 examined to date, most induce the proteasomal degradation of one or more of the IRF proteins (IRF3, IRF5, and IRF7) involved in stimulating IFN expression. However, there are exceptions such as OSU NSP1, which blocks IFN-β induction by inducing the degradation of β-TrCP, a protein that is necessary for NF-κB activation. It is important to note that our current understanding of NSP1 activities is based chiefly on the study of relatively few strains of cell culture-adapted animal RVs. Thus, we may have a somewhat skewed perspective of what is typical for NSP1. Moreover, it is possible that the design of the assay systems used in testing the activities of NSP1 may have introduced biases into experimental results. For example, whether an NSP1 protein can degrade an IRF target in an assay system may depend not only on the viral source of NSP1, but also on the species origin of the target.

NSP1 has been established to interact with several host proteins, including those in the IRF family and β-TrCP. It is quite likely that the function of NSP1 requires it to interact with other host proteins as well, mostly notably the E2 ligases. It is possible that failures noted in the ability of some NSP1 proteins to degrade target proteins may not stem from the inability of NSP1 to physically engage a target, but may instead be due to the failure of an E2 ligase to interact successfully with an NSP1-target protein complex. Certainly, the lack of compatibility between the host E2 ligase and NSP1 could be a defining factor in RV host range restriction and cell tropism. Also unresolved is the relative importance of the strength and stability of the interaction between NSP1 and its target as a factor in target degradation.

Currently, no single sequence feature of NSP1 has been identified that can be tied to the ability of some RV strains but not others to degrade IRF3. Phylogenetic analyses rules out any obvious link between the activities of NSP1 and genotype of the protein, or the serotype or host range of the virus encoding the protein. Studies of the NSP1 activities of a diverse collection of RV isolates have been slowed by difficulties raising hyperimmune serum against NSP1, reflecting the low antigenicity of the protein [81]. Additionally, the few antibodies that have been generated to recognize NSP1 are exquisitely specific, with little to no capacity to cross-react with NSP1s from different RV species. The development of antibodies to several strains of RV NSP1 or a single highly cross-reactive antibody would greatly enhance future studies of the protein and its activities.

An important unresolved question is whether the RNA-binding activity of NSP1 has a role in suppressing IFN induction. One possibility is that NSP1 may use this activity to sequester viral RNAs in infected cells, similar to the IFN-antagonist protein of influenza A virus NS1, thereby preventing interactions with PRRs [109–111]. Some evidence indicates that RV infection might stimulate innate immune responses through recognition of viral RNAs by RIG-I [112]. However, the association of NSP1 with PRRs upstream of IRF3 remains unexplored.

NSP1 plays a role in RV pathogenesis as a broad-spectrum antagonist of IFN induction, yet many unanswered questions remain. Are there other cellular targets degraded or otherwise targeted for inactivation by NSP1? Does the RNA-binding activity of NSP1 play a role in subverting the IFN response? What are the sequence determinants of different NSP1 activities? Is NSP1 an E3 ubiquitin ligase, and if so, what is its E2 partner? Does NSP1 from human strains of RV share the activities that have been described for the cell culture-adapted animal strains? The answers to such questions will lend insight into how NSP1 enhances virulence of RV in infected hosts. There are several challenges to studying RV NSP1, including difficulties in adapting and growing human RVs in cell culture, the expense and relative lack of large animal models, and the absence of an appropriate reverse genetics system. However, developing solutions to these challenges will enhance and renew efforts to create new and more effective vaccine candidates.

## Figures and Tables

**Table 1.** Capacity of NSP1 from RV strains shown to degrade endogenous IRF3.

RV Strain	Host Species	IRF3 Degradation (Cell Type Tested)	Reference
SA11-4F	Simian	+ (Caco-2, FRhL_2_, MA104)	[86,89,90]
30–19	Simian	+ (FRhL_2_)	[89]
RRV	Simian	+ (Cos7, MEF)	[91,92]
UK	Bovine	+ (Cos7) − (MEF)	[91,92]
B641	Bovine	+ (MA104)	[90]
NCDV	Bovine	+ (MA104, HEK293) − (MEF)	[90,92]
OSU	Porcine	− (MA104, HEK293)	[90,92]
ETD	Murine	+ (Cos7, MEF)	[91,92]
EW	Murine	+ (MEF)	[91]
